# Transmembrane Domain—Dependent Functional Oligomerization of Syndecans

**DOI:** 10.1100/tsw.2006.92

**Published:** 2006-04-07

**Authors:** Jae Youn Yi, Innoc Han, Eok-Soo Oh

**Affiliations:** ^1^ 1Laboratory of Tissue Engineering, Korea Institute of Radiological and Medical Sciences, Korea Atomic Energy Research Institute, Seoul 120-725, Republic of Korea; ^2^ College of Medicine, Department of Physiology and Biophysics, Inha University, Incheon 402-751, Republic of Korea; ^3^ Department of Life Sciences Center for Cell Signaling Research, Ewha Womans University, Seoul 120-725, Republic of Korea

**Keywords:** syndecan, heparan sulfate proteoglycan, receptor, transmembrane domain, dimerization, oligomerization, receptor activation

## Abstract

Cell surface adhesion receptors of the syndecan family initiate intracellular events through clustering of receptors. This crucial clustering occurs through receptor dimerization or oligomerization, which is mediated by receptor transmembrane domains. However, the exact role of the transmembrane domain during receptor activation is not fully understood. Researchers have not yet determined whether the transmembrane domain functions solely in the physical aspects of receptor clustering, or whether the domain has additional functional roles. Here we review recent advances in understanding the functionality of transmembrane domain—dependent oligomerization of syndecan cell adhesion receptor.

Living cells must sense and specifically respond to changes in the environment, which is predominantly formed by the surrounding extracellular matrix (ECM). A variety of factors can serve as signals, including physical factors such as adhesion of cells to the ECM[1] and chemical factors such as growth factors [e.g., epidermal growth factor (EGF) and platelet-derived growth factor (PDGF)][2]. Regardless of the nature of stimuli, cells typically sense and respond through cell surface transmembrane receptor proteins that are capable of transmitting signals from the extracellular environment to the intracellular compartment on ligand binding. The binding of a ligand induces conformational changes in the receptors, inducing them to cluster together in a process known as receptor dimerization or oligomerization[2,3]. This dimerization/oligomerization, in turn, triggers changes in various cellular processes. For example, dimerization of most growth factor receptors activates their kinase activity, leading to phosphorylation of the receptors at numerous intracellular tyrosine or serine/threonine residues. This phosphorylation helps to recruit various signaling molecules, which are activated via phosphorylation and subsequently released to affect numerous cellular events. Numerous studies have shown that the transmembrane domains of receptor proteins are vital to receptor clustering[4,5,6]. Thus, it is important that we fully understand the mechanisms of receptor dimerization and the role of the transmembrane domain in controlling cellular signal transduction.

The syndecans comprise a family of transmembrane cell surface heparan sulfate proteoglycans that localize to the plasma membrane and function as adhesion receptors, binding the ECM and/or soluble ligands and subsequently regulating adhesion-dependent intracellular signal transduction[7,8]. Similar to classical growth factor receptors, syndecans exhibit a propensity to form noncovalently linked dimers and/or oligomers. All members of this family form homologous dimers or multimers that are strong enough to resist dissociation by SDS treatment[9,10]. In 1995, Asundi and Carey[10] showed that recombinant syndecan-3 core protein formed stable multimer complexes, and that this multimerization required the transmembrane domain and a short sequence in the ectodomain flanking region. Similarly, our group showed that recombinant syndecan-2 and syndecan-4 core proteins lacking the cytoplasmic domain could form SDS-resistant oligomers[11]. These studies indicate that the transmembrane domain plays a crucial role in syndecan oligomerization. However, the importance of the transmembrane domain in overall syndecan function is less well understood.

To better understand syndecan oligomerization, it is helpful to draw parallels with other receptors, including integrin adhesion receptors and growth factor receptors, such as EGF receptor (EGFR), PDGF receptor (PDGFR), and transforming growth factor β receptor (TGFβR)[1,12,13,14]. In the absence of ligand binding, integrins and TGFβR exist as heterodimers composed of αβ and typeI/typeII heterodimers or oligomers, respectively[1,12]. After ligand binding, these receptors undergo activating conformational changes. In contrast, unbound PDGFR and EGFR exist singly, and ligand binding triggers activating homo- and/or hetero-dimerization events[13,14]. Interestingly, recent studies have shown that receptor dimerization or oligomerization is mediated by specific, potentially functional protein regions of these receptors. For example, the extracellular domain plays a crucial role in EGFR dimerization[14], while the transmembrane domain of TGFβR plays a pivotal role in its activation[12]. In all of these cases, however, the transmembrane domain undergoes ligand-induced conformational changes required for receptor activation. Thus, it is tempting to speculate that the transmembrane domain may be responsible for regulating specific receptor functions.

It is not yet clear whether transmembrane domains function simply in a physical manner, i.e., by clustering receptors and bringing their cytoplasmic domains into close proximity with one another, or if this domain plays a more active functional role in some or all receptors. Choi et al.[15] showed that the transmembrane domains of syndecan-2 and syndecan-4 are sufficient for inducing oligomerization, and that transmembrane domain–induced oligomerization is crucial for the functions of these receptors. Specifically, syndecan-2 and -4 constructs containing the transmembrane domain formed SDS-resistant dimers, but constructs lacking the transmembrane domain did not. When the syndecan transmembrane domains were substituted with that of PDGFR, forming syndecan/PDGFR chimeras, the authors observed very few SDS-resistant dimers, indicating that the transmembrane domains were receptor specific for oligomerization. Furthermore, although wild-type PDGFR dimerizes through its transmembrane domain and undergoes dimerization-dependent activation, the syndecan/PDGFR chimeras lost their specific signaling functions; the syndecan-2/PDGFR chimera was unable to induce cell migration, while the syndecan-4/PDGFR chimera was incapable of mediating focal adhesion formation and activation of protein kinase C. These results indicate that the transmembrane domain–induced oligomerization of syndecans is specific and required for receptor function, strongly suggesting that the transmembrane domains of these receptors have *in vivo* functions beyond simple physical clustering. In general, the activation of downstream signaling is dependent on the oligomeric status of the cytoplasmic domain of receptors during transmission of signals to the intracellular environment. The cytoplasmic domains of receptors require proximity but not necessarily oligomerization for their actions. Thus, the transmembrane domain inevitably regulates the activity of the cytoplasmic domain by driving oligomerization, perhaps accounting at least in part for the apparent regulatory functions of the transmembrane domains in syndecan receptor proteins.

In sum, numerous studies have shown that the transmembrane domain potentially regulates the activation and function of syndecan receptor proteins. Future work will be required to fully define how transmembrane-dependent oligomerization confers the functional uniqueness of receptors, and whether this phenomenon is generalizable to all cell surface receptors. However, recent advances in our understanding of syndecan multimerization and activation have provided new insights into the mechanisms by which cells sense and respond to their environments.

## ACKNOWLEDGMENTS

This work was supported by Korea Research Foundation Grant (KRF-2002-CP0327 to E.S.O) and Korea Science and Engineering Foundation (KOSEF) through the Center for Cell Signaling Research at Ewha Womans University.
